# Explaining oscillations and variability in the p53-Mdm2 system

**DOI:** 10.1186/1752-0509-2-75

**Published:** 2008-08-18

**Authors:** Carole J Proctor, Douglas A Gray

**Affiliations:** 1 Centre for Integrated Systems Biology of Ageing and Nutrition, Institute for Ageing and Health, Newcastle University, Newcastle upon Tyne, NE4 5PL, UK; 2 Ottawa Health Research Institute, Ottawa, ON K1H 8L6, Canada; 3 Department of Biochemistry, Microbiology and Immunology, University of Ottawa, Ottawa, ON K1H 8M5, Canada

## Abstract

**Background:**

In individual living cells p53 has been found to be expressed in a series of discrete pulses after DNA damage. Its negative regulator Mdm2 also demonstrates oscillatory behaviour. Attempts have been made recently to explain this behaviour by mathematical models but these have not addressed explicit molecular mechanisms. We describe two stochastic mechanistic models of the p53/Mdm2 circuit and show that sustained oscillations result directly from the key biological features, without assuming complicated mathematical functions or requiring more than one feedback loop. Each model examines a different mechanism for providing a negative feedback loop which results in p53 activation after DNA damage. The first model (ARF model) looks at the mechanism of p14^ARF ^which sequesters Mdm2 and leads to stabilisation of p53. The second model (ATM model) examines the mechanism of ATM activation which leads to phosphorylation of both p53 and Mdm2 and increased degradation of Mdm2, which again results in p53 stabilisation. The models can readily be modified as further information becomes available, and linked to other models of cellular ageing.

**Results:**

The ARF model is robust to changes in its parameters and predicts undamped oscillations after DNA damage so long as the signal persists. It also predicts that if there is a gradual accumulation of DNA damage, such as may occur in ageing, oscillations break out once a threshold level of damage is acquired. The ATM model requires an additional step for p53 synthesis for sustained oscillations to develop. The ATM model shows much more variability in the oscillatory behaviour and this variability is observed over a wide range of parameter values. This may account for the large variability seen in the experimental data which so far has examined ARF negative cells.

**Conclusion:**

The models predict more regular oscillations if ARF is present and suggest the need for further experiments in ARF positive cells to test these predictions. Our work illustrates the importance of systems biology approaches to understanding the complex role of p53 in both ageing and cancer.

## Background

**Figure 1:**
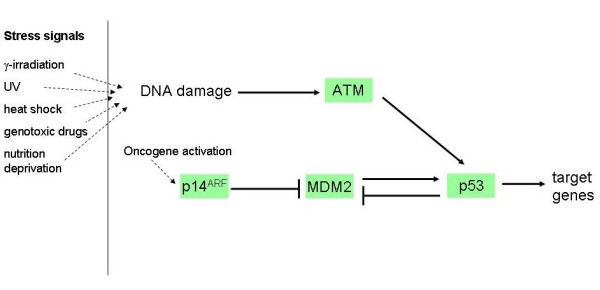
Network diagram of the p53 signalling pathway. Adapted from the KEGG signalling pathway [11].

The p53 tumour suppressor plays a major role in cancer, as evidenced by frequent *TP53 *gene mutations in human tumours [1] and by the high incidence of cancer in Li-Fraumeni individuals carrying germline mutations in the *TP53 *gene [2]. There is a growing consensus that p53 plays an important role in ageing and limitations to lifespan (reviewed in Bauer and Helfand (2006) [3] and in Papazoglu and Mills (2007) [4]) but this assertion has been recently challenged on evolutionary grounds [5]. The *TP53 *gene encodes a transcription factor with target genes that are involved in DNA repair, cell cycle arrest and apoptosis. It has been described as the 'guardian of the genome' [6], blocking cell cycle progression to allow the repair of damaged DNA. It has also been described as a 'gatekeeper' [7-9] that dictates the fate of cells that have suffered stress by directing them into irreversible pathways of senescence or apoptosis [10]. Figure 1 shows part of the p53 signalling pathway which is a simplification of the KEGG pathway [11]. Under normal homeostatic conditions the cellular levels of p53 protein are kept at a low level. There is basal transcription of the p53 gene even in unstressed cells but the protein product does not accumulate as it has a short half-life (about 15–30 minutes) [12] and is usually bound to Mdm2, an ubiquitin E3 ligase, which targets p53 to the proteasome for degradation [13,14]. Mdm2-binding prevents the transcriptional activity of p53 [15], a phenomenon that is dependent on the catalytic activity of Mdm2 [16]. Mdm2 also has a short half-life and is a substrate of its own E3 ligase activity in vitro [17]. The degradation of a knocked-in RING finger mutant of Mdm2 indicates the presence of an as-yet unidentified cellular E3 ligase that ubiquitinates Mdm2 in vivo [18]. The transcription of Mdm2 is regulated by p53 [19] and so under normal conditions, levels of both p53 and Mdm2 are kept at low levels.

**Figure 2:**
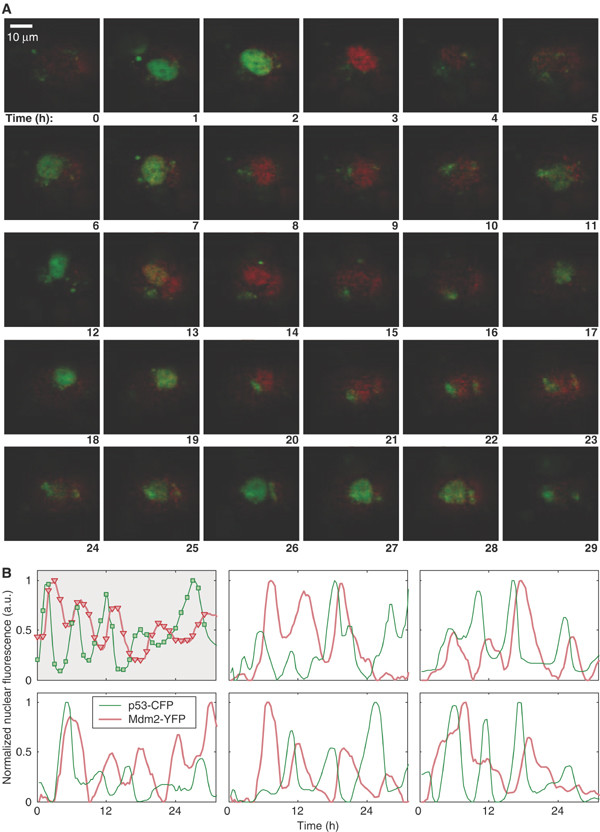
Prolonged oscillations in the nuclear levels of fluorescently tagged p53 and Mdm2 in individual MCF7, U280 cells following gamma irradiation. Reproduced with permission from Geva-Zatorsky et al. 2006, Molecular Systems Biology [30]. A. Time-lapse fluorescence images of one cell over 29 h after 5 Gy of gamma irradiation. Nuclear p53-GFP and Mdm2-YFP are imaged in green and red, respectively. Time is indicated in hours. B. Normalised nuclear fluorescence levels of p53-CFP (green) and Mdm2-YFP (red) following gamma irradiation. Top left: the cell shown in panel A. Other panels: five cells from one field of view, after exposure to 2.5 Gy gamma irradiation.

It is well known that stress induces an increase in levels of p53 which in turn leads to an increase in the transcription of Mdm2 [20]. One pathway for stabilization of p53 is via the kinase ATM, which is activated by DNA damage and phosphorylates p53 close to its Mdm2 binding site, so blocking its interaction with Mdm2 [9]. In addition, ATM phosphorylates Mdm2 which not only interferes with its ability to bind to p53 but also enhances the degradation of Mdm2 [21,22] providing an additional route for p53 stabilization. Another mechanism for the increase in p53 levels is the activation of ARF (known as p14^ARF ^in humans), a nucleolar protein that senses DNA damage [23]. It has been shown that DNA damage disrupts the interaction of ARF with the nucleolar protein B23 (nucleophosmin) releasing ARF into the nucleoplasm so that it can bind to Mdm2 [24]. ARF binding enhances the degradation of Mdm2, resulting in p53 stabilisation [23,25]. ARF also responds to aberrant growth signals which are triggered by oncogenes such as Ras or Myc, although the induction of genes from the INK4a/ARF locus displays species-specific variations [26]. Since an increase in p53 leads to an increase in Mdm2 transcription, and Mdm2 targets p53 for degradation, p53 levels are again inhibited, providing a negative feedback loop. Negative feedback loops have been found in several systems of interacting proteins (e.g. Hes1 in Notch signalling [27], NF-kB signalling system [28]) and have attracted the attention of mathematical modellers. In particular, models have been produced to analyse the oscillations of p53 and Mdm2 in previously published single-cell fluorescent reporter assays [29-34]. The single cell assays have been very informative, revealing that increasing DNA damage results in an increased number of oscillations, but not an increased magnitude in the response [30,35]. The data also show that there is large intercellular variation with a fraction of cells showing no response or a slowly fluctuating signal. In the cells in which oscillations were detected, there was a wide fluctuation in the amplitude (about 70%) and smaller variations in the period of the peaks (about 20%) [30]. The oscillations in these data showed a period of about 5.5 hours with a delay of about 2 hours between p53 and Mdm2 peaks (Figure 2, which is a reproduction of figure 1 from Geva-Zatorsky et al. [30] with permission from the publisher).

All previous models to date have used a deterministic approach to analyse the oscillatory behaviour. These models have used differential equations and mathematical functions requiring a fairly large number of parameters with the generation of oscillations being very dependent on the range of parameter values chosen. Geva-Zatorsky et al. [30] constructed six different models and found that the simplest model, which contained one intermediary and one negative feedback loop with a delay, was unable to produce multiple oscillations and that it was necessary to either introduce a positive feedback loop or a time delay term (See figure 6 of Geva-Zatorsky et al. [30]). However, these additions were not sufficient for robustness over a wide range of parameter values. The addition of a non-linear negative feedback loop, a linear positive feedback loop or a second negative feedback loop produced models that were able to demonstrate sustained oscillations over a wide range of parameters. As the models are deterministic, the outcome only depends on the initial conditions and so they cannot be used to investigate cell-cell variability. Geva-Zatorsky et al. [30] incorporated some random noise in protein production in their models and found that the introduction of low-frequency noise resulted in variability in the amplitude of the oscillations as observed experimentally. Ma et al. [32] also incorporated a stochastic component for the DNA damage component of their model which resulted in variability in the number of oscillations. However, for a dose of 2.5 Gy, they found that the majority of cells had only one peak and that a step input of DNA damage was required to obtain sustained oscillations.

The deterministic models have been useful in showing that sustained oscillations can be produced in a system where there is at least one negative feedback loop with a delay, and a sustained signal. The signal represents damaged DNA which triggers the cellular response as long as the DNA damage persists [36]. It has also been shown that stochasticity in protein production rates and DNA damage events can explain some of the variability in the data. However, in cellular systems, there will be random effects on all processes. Most of the previous models ignored the fact that p53 has to bind to Mdm2 for its Mdm2-dependent degradation and that it is transcriptionally inactive when bound. Instead, the models assumed that p53 degradation depended on total Mdm2 levels regardless of whether Mdm2 was bound to p53 or not. Since the regulation of p53 is dependent on its interaction with Mdm2, we would expect that the oscillatory behaviour of the system would be strongly affected by the binding affinity of Mdm2 to p53. Therefore any mechanistic model of the system should include the Mdm2-p53 complex.

Other disadvantages of the current models are that they cannot be easily modified or linked to other models and they are not very accessible to non-mathematicians. Most importantly, the majority of previous models have not clearly demonstrated how the biological mechanisms of the system contribute to the oscillatory behaviour. The deterministic models of Ma et al. 2005 [32], Ramalingam et. al 2007 [37] and Ciliberto et al 2005 [29] are based on molecular mechanisms but none of these are really suitable for a stochastic approach, since the Gillespie algorithm assumes mass action kinetics and these models contain Hill or Michaelis-Menten functions in their rate laws. We chose to build the simplest possible stochastic model using a mechanistic approach that would be particularly relevant to biologists (see Figure 2 and Methods).

The aims of building a stochastic mechanistic model are three-fold. First, we wanted to see if a simple model with stochastic effects would produce sustained oscillations without the need to introduce additional feedback loops or non-linear functions. Second, we wanted the model to be based on the mechanisms that have been proposed by biologists and could be easily understood by the non-mathematically inclined. Third, we wanted to build a model that can be easily incorporated into a larger model such as our earlier model of the ubiquitin-proteasome system [38]. In order to achieve these objectives we used the Systems Biology Markup Language (SBML) [39]. SBML is a well-known modelling standard, allowing models to be shared in a form that other researchers can use even in a different software environment. Since both ATM and ARF activation have been proposed as mechanisms for stabilising p53 after DNA damage, we developed two independent models to see whether oscillations would result from either of these mechanisms. The ARF model is simpler, and so we introduce this model first and show how it can be modified to produce the ATM model.

There are many tools available for creating and running SBML models (see ). We chose to use the Biology of Ageing e-Science Integration and Simulation system (BASIS) [40,41] to store the models, run simulations and store results. The advantage of this system is that it is user friendly, it can be freely accessed by a web browser, and allows easy sharing of models.

## Results

### (1) ARF Model

#### Small oscillations are not distinguishable from white noise under normal conditions

**Figure 3:**
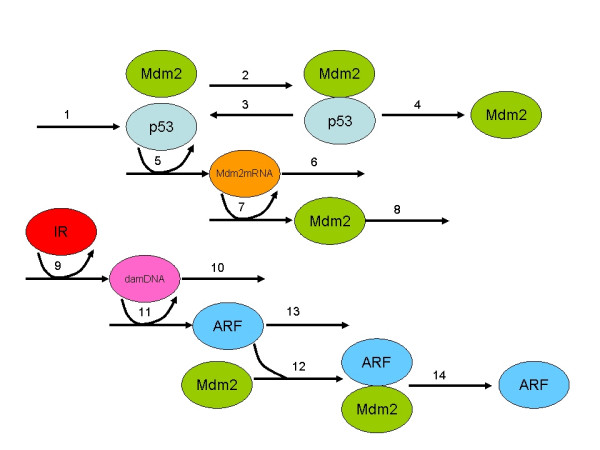
Diagram of the system for the ARF model. Details of the reactions are in Table 2. IR is activated under conditions of irradiation at time t = 1 hour and then deactivated again 1 minute later. The numbers on the arrows refer to the reaction numbers in Table 2.

**Figure 4:**
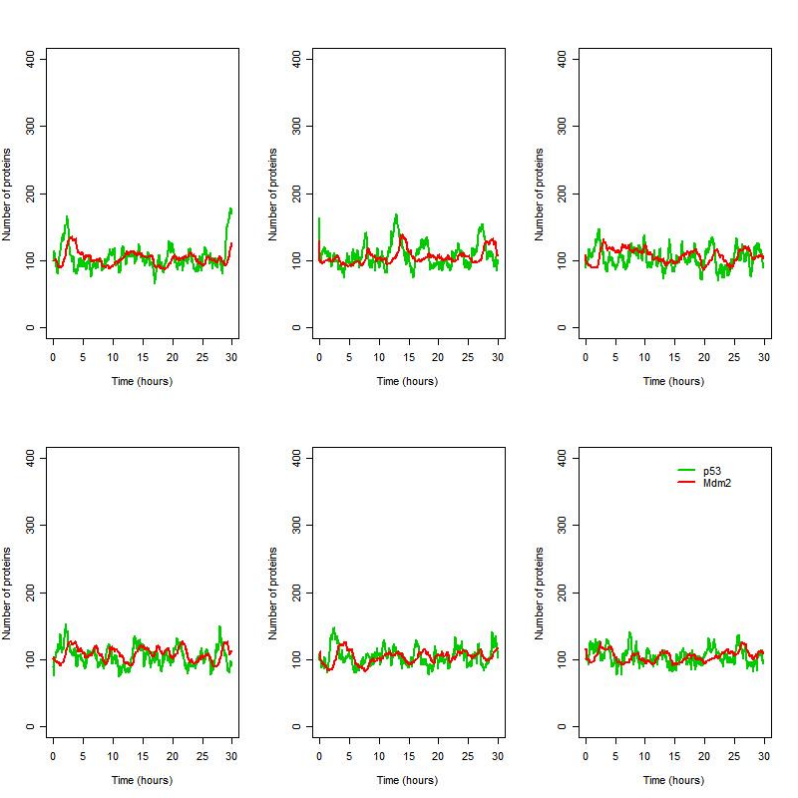
Six simulations of the ARF model under normal conditions (no irradiation).

**Figure 5:**
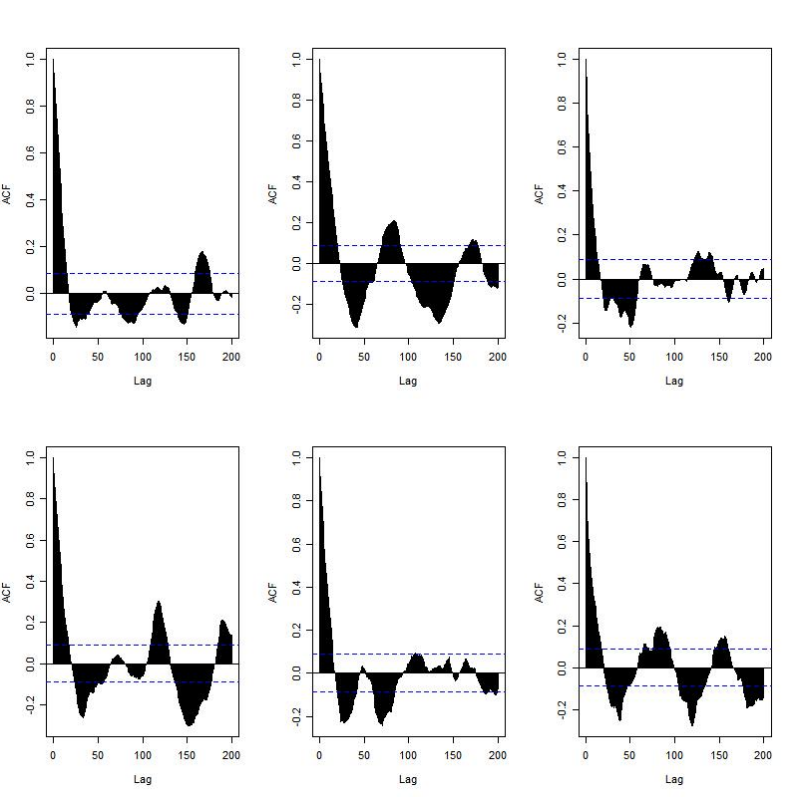
The p53 autocorrelation function (ACF) for the six simulations under normal conditions shown in Figure 4. (The ACF for Mdm2 was very similar and so not shown.)

The full list of species and reactions for the ARF model are listed in Tables 1 and 2 respectively. Figure 3 shows a diagram of the system (full details are given in the methods section). Even under normal conditions, there is synthesis and degradation of both p53 and Mdm2 so that we might expect low level oscillations of both proteins. However, since we have used a stochastic simulator, there is also a large component of white noise due to protein synthesis and degradation being modelled as random processes and this would mask any oscillatory effects (Figure 4). The autocorrelation function (ACF) for p53 was computed and plotted using the R statistical package. A periodic ACF is consistent with oscillations whereas a non-periodic ACF is consistent with noise. The ACF confirms that most of the oscillatory behaviour in the model is due to white noise but there are some regular oscillations in the second simulation (see Figure 5, top row, 2^nd ^column). The autocorrelation function for Mdm2 showed similar behaviour and so the plots are not shown.

**Table 1:** List of species for the ARF model

Name	Description	Database term	Initial amount
p53	Unbound p53 protein	P04637	5
Mdm2	Unbound Mdm2 protein	Q00987	5
Mdm2_p53	Mdm2/p53 complex	P04637/Q00987	95
Mdm2_mRNA	Mdm2 messenger RNA	SBO:0000278	0
ARF	Unbound ARF protein	Q8N726	0
ARF_Mdm2	ARF/Mdm2 complex	Q8N726/Q00987	0
IR	Dose of irradiation	n/a	0 dGy
damDNA	Amount of damaged DNA	CHEBI16991	0

Terms starting with:

P or Q are from UniProtKB/Swiss-Prot [61].

SBO are from Systems Biology Ontology [62]

CHEBI are from Chemical Entities of Biological Interest Database [63]

**Table 2:** List of reactions for the ARF model

Reac No.^a^	Name	Term	Kinetic law	Parameter values^b^	
1	p53 synthesis	GO:0006412	*k*_*synp*53_	7.8E-2 mol s^-1^	Based on p53 half-life ~20 min [12].
2	p53/Mdm2 binding	GO:0002039	*k*_*binMdm*2*p*53_<#p53><#Mdm2>	1.155E-3 mol^-1 ^s^-1^	Assumed that 95% of p53 is bound to Mdm2 under normal conditions.
3	Mdm2_p53 dissociation	GO:0043624	*k*_*relMdm*2*p*53 _<#Mdm2_p53>	1.155E-5 s^-1^	Based on dissociation constant of ~100 molecules [56].
4	p53 degradation	GO:0043161	*k*_*degp*53_<#Mdm2_p53>	8.25E-4 s^-1^	p53 half-life ~20 min [12].
5	Mdm2 Transcription	GO:0003700	*k*_*synMdm*2*mRNA*_<#p53>	1.0E-4 s^-1^	Turnover of Mdm2_mRNA was adjusted to give period of oscillations ~5–6 h [35].
6	Mdm2_mRNA degradation	GO:0006402	*k*_*degMdm*2*mRNA*_<#Mdm2_mRNA>	1.0E-4 s^-1^	
7	Mdm2 synthesis	GO:0006412	*k*_*synMdm*2_<#Mdm2_mRNA>	4.95E-4 s^-1^	Based on Mdm2 half-life ~30 min [12].
8	Mdm2 degradation	GO:0043161	*k*_*degMdm*2_<#Mdm2>	4.33E-4 s^-1^	Mdm2 half-life ~30 min [12].
9	DNA damage	GO:0006974	*k*_*dam*_<#IR>	8.0E-2 s^-1^	Based on about 30 double strand breaks per cell per Gy irradiation [64].
10	DNA repair	GO:0006281	*k*_*repair*_<#damDNA>	2.0E-5 s^-1^	This was set so that all DNA damage was repaired by ~10–16 h[65].
11	ARF activation	GO:0030330	*k*_*actARF*_<#damDNA>	3.3E-5 s^-1^	ARF is activated within 1 hour of DNA damage and activity peaks at 6 h.
12	ARF/Mdm2 binding	GO:0005515	*k*_*binARFMdm*2_<#ARF><#Mdm2>	1.0E-2 mol^-1 ^s^-1^	This was set so that ARF/Mdm2 complexes peaks at 6–8 h after IR [23].
13	ARF degradation	GO:0043161	*k*_*degARF*_<#ARF>	1.0E-4 s^-1^	Based on ARF half-life ~6 h [66].
14	ARF-dependent Mdm2 degradation	GO:0043161	*k*_*degARFMdm*2_<# ARF_Mdm2>	1.0E-3 s^-1^	Assumed to be 3-fold faster than normal degradation 7[25].

^a^Reaction numbers correspond to the numbered arrows in Figure 3, ^b^mol refers to the number of molecules

#### One intermediary in a negative feedback loop and sustained damage is sufficient to produce oscillatory behaviour

**Figure 6:**
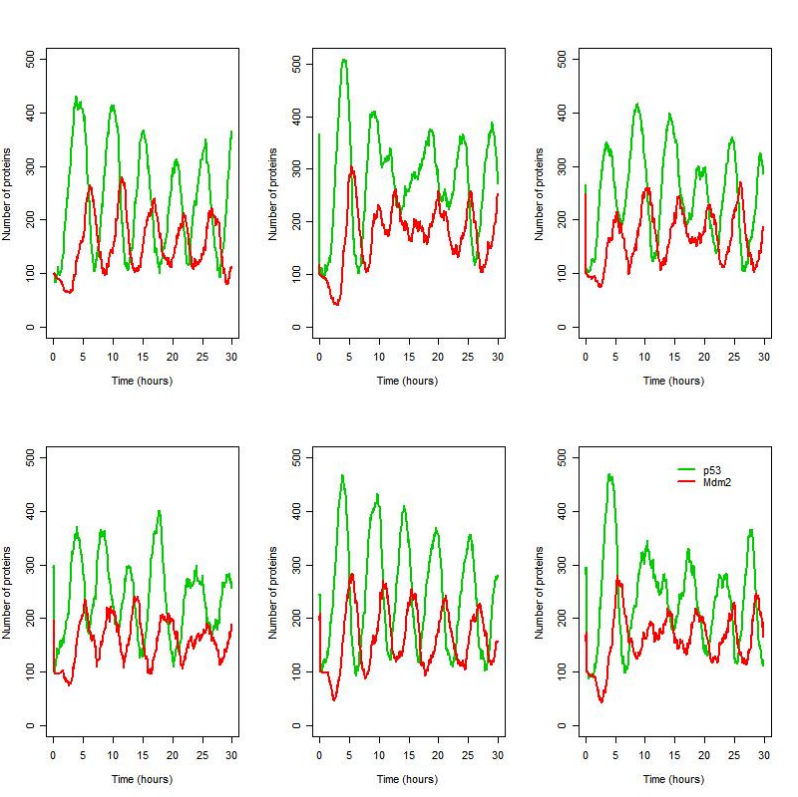
Six simulations for the ARF model under conditions of irradiation (IR = 25 dGy for 1 minute at time t = 1 hour).

**Figure 7:**
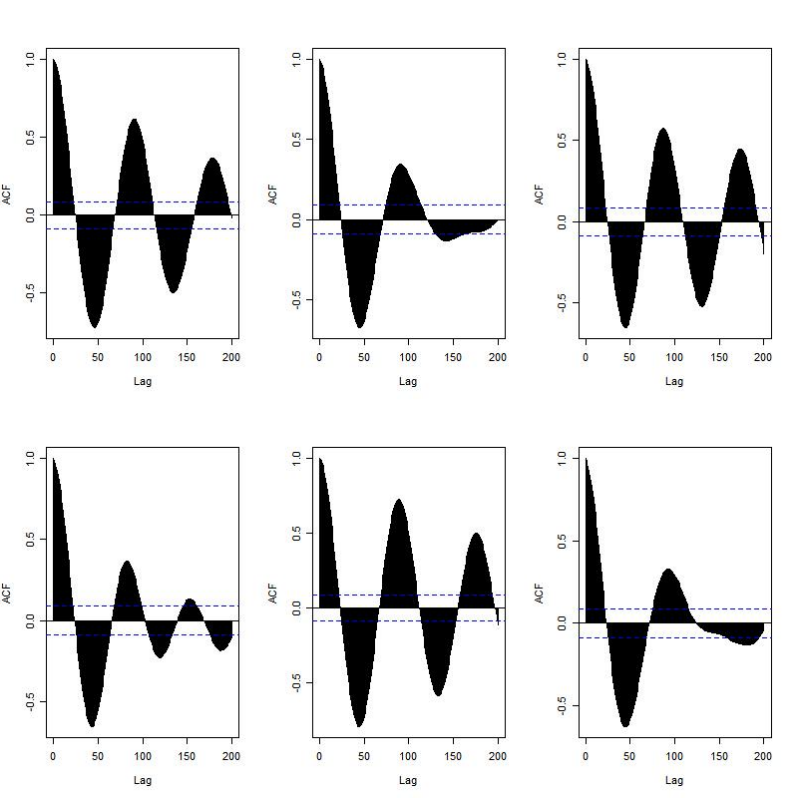
The p53 autocorrelation function (ACF) for the six simulations of irradiated cells as shown in Figure 6. (The ACF for Mdm2 was very similar and so not shown.)

We started by building the model without any intermediary species so that Mdm2 synthesis only depended on the level of unbound p53. This model did not predict sustained oscillations after induction of DNA damage (data not shown). The addition of one intermediary, Mdm2_mRNA, in the negative feedback loop was sufficient for the appearance of sustained oscillations, in both p53 and Mdm2, confirming that a delay is required (Figure 6). Mdm2_mRNA represents the messenger RNA molecule which is transcribed from the Mdm2 gene and carries the coding information to the site of protein synthesis. The autocorrelation function shows that these oscillations are distinct from those generated by white noise (Figure 7). The model output shows that there is intercellular variability as seen in the data. In particular, although most simulations resulted in 5 or 6 peaks in a 30 hour period, there were occasional simulated "cells" with 4 or less peaks. There is also intracellular variability in the oscillation amplitude but much less variation in the oscillation period which was about 5.5 hours. The model predictions regarding the oscillation period agrees with the experimental data [30] as expected, since the parameters which affect the period are well known (see Table 3). The agreement of the model predictions with the data, regarding intracellular and intercellular variability, confirms that a stochastic approach is appropriate for this system.

**Table 3:** Summary of sensitivity analysis for the ARF model

Biological process	Parameters changed	Effect on period as parameter is increased (decreased)		Effect on amplitude as parameter is increased (decreased)	
		× 2	× 10	× 2	× 10

p53 turnover	*k*_*synp*53_, *k*_*degp*53_	↓ (↑)	↓ (oscillations lost)	↑ (↓)	↑ (oscillations lost)
Mdm2 turnover	*k*_*synMdm*2_, *k*_*degMdm*2_	↓ (↑)	oscillations lost (↑)	↓ (↑)	oscillations lost (↓)
Mdm2_mRNA turnover	*k*_*synMdm*2*mRNA*_ *k*_*degMdm*2*mRNA*_	↓ (↑)	↓ (↑)	↓ (↑)	↓ (↑)
p53/Mdm2 binding and release	*k*_*binMdm*2*p*53_, *k*_*relMdm*2*p*53_	↔ (↔)	↔ (↔)	↓ (↑)	↓ (↑)
DNA damage	*k*_*dam*_	↔ (↔)	↔ (↔) (Greater variability in period for ×10 increase)	↔ (↔)	↑ (↓) (Very few cells with oscillations for ×10 decrease)
DNA repair	*k*_*repair*_	↔ (↔)	↔ (↔)	↓ (↑)	↓ (↑) (Very few cells with oscillations for ×10 increase)
ARF activation	*k*_*actARF*_	↔ (↔)	↔ (↔)	↑ (↓)	↑ (↓) (Very few cells with oscillations for ×10 decrease)
ARF degradation	*k*_*degARF*_	↔ (↔)	↔ (↔)	↔ (↔)	↓ (↑)
ARF/Mdm2 binding	*k*_*binARFMdm*2_	↔ (↔)	↔ (↔)	↔ (↓)	↔ (↓)
ARF-dependent Mdm2 degradation	*k*_*degARFMdm*2_	↔ (↔)	↔ (↔) (Period is very irregular in both cases)	↔ (↓)	↔ (↓)

Key

↔ No change, ↓ Decrease in period or amplitude, ↑ Increase in period or amplitude

#### Predictions when the efficiency of protein degradation is reduced

Since it is known that proteasomal activity declines with age [42], and in tissues such as the rodent cerebral cortex, the age-related loss of chymotryptic proteasomal activity is significant (40%, as reported by Zeng et al [43]), we decided to test the effect of reduced degradation by scaling all the parameters for protein degradation by the same factor (*k*_*degp*53_, *k*_*degMdm*2_, *k*_*degARF*_, *k*_*degARFMdm*2_). The model predicted that decreasing protein degradation efficiency by up to 30% had little effect, but larger decreases resulted in lengthening the oscillation period. If protein degradation efficiency was less than 50%, then the levels of both Mdm2 and p53 increased rapidly after DNA damage and the values oscillated about a mean value of about 350 for p53 and 400 for Mdm2 (instead of about 250 and 200 respectively), although the amplitude of the peaks was similar (data not shown).

#### Effect of changing the amount of damage

To simulate the effect of varying the dose of gamma irradiation, we changed the level that the species IR was increased after the irradiation event in the model. The default value of 25 dGy corresponds to a dose of 2.5 Gy. We used units of dGy, rather than Gy, in our model, as it is necessary to use integer numbers for stochastic simulation. The effect of increasing this to 50 dGy (i.e. 5 Gy) had no effect on the oscillation period and little effect on the amplitude, however there were no cells with less than 4 peaks, as was the case in the default model. Conversely, decreasing the value of IR after irradiation led to fewer cells with oscillations. Therefore our model agrees with the experimental observation that increasing irradiation dose produces more peaks but does not affect the oscillation period. However, our model predicts a slight decrease in the amplitude of the peaks at low irradiation doses (IR = 1 dGy).

#### Effect of a gradual increase in DNA damage from oxidative stress

**Figure 8:**
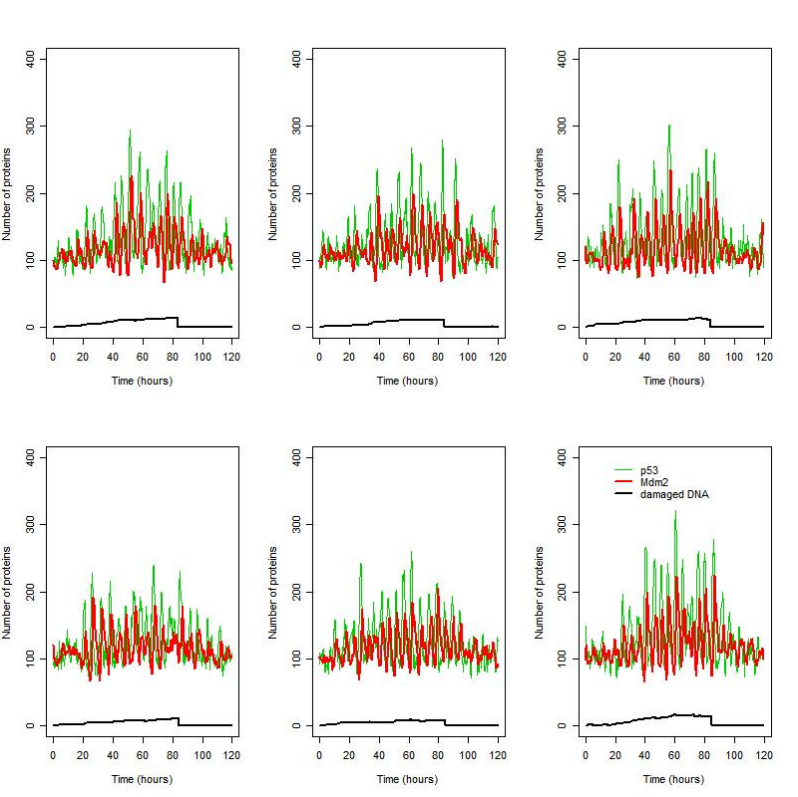
Oscillations are triggered when DNA damage accumulates gradually over time and disappear when DNA is repaired (ARF model). DNA damage starts to accumulate at time t = 0, and increases until t = 40 hours, when the rate of DNA repair is increased ten thousand-fold.

During ageing there is a gradual increase in damaged DNA due to either an increase in oxidative stress and/or a decrease in anti-oxidant capacity. We used our model to see if oscillations would occur in this scenario. To simulate the effect of a gradual increase in DNA damage we introduced a species called ROS (reactive oxygen species) into the model and changed the reaction for DNA damage so that it depended on the level of ROS instead of IR. We set the rate law for DNA damage reaction so that it gradually increased over time. We also added an event at time 40 hours to increase the repair capacity to see if the oscillations would die down after DNA damage is repaired. The model predicts that oscillations appear when DNA damage starts to accumulate and then remain even after DNA damage is repaired, although the amplitude decreases over time (Figure 8). The persistence of the oscillations is due to the slow turnover of ARF. If we increase the degradation rate of ARF, then the oscillations die out more quickly (data not shown). Our simulations suggest that it is not necessary to have a sudden increase in damage in order to trigger spontaneous oscillations.

#### Effect of varying model parameters

We varied each of the model parameters to test the robustness of the model and to see which parameters affected the oscillatory behaviour. The results are summarised in Table 3. The model is robust to small changes in the parameter values (either two-fold decrease or two-fold increase) and even ten-fold changes in some of the parameters did not result in loss of oscillatory behaviour. The changes which did result in loss of oscillations were in the parameters that affected DNA damage, either because there was not enough damage to produce oscillations, or it was removed too quickly to sustain the damage signal. An increase in the turnover rate of p53, Mdm2 or Mdm2_mRNA (the intermediary) resulted in a reduction in the oscillation period. Conversely, a decrease in these parameters resulted in an increase in the oscillation period. No other parameters had any significant effect on the period. We conclude that the oscillation period is very dependent on the turnover rates of p53 and Mdm2. An increase in the turnover of p53 resulted in an increase in the oscillation amplitude, whereas an increase in the turnover of Mdm2 or Mdm2_mRNA, or in the binding affinity of Mdm2 to p53 resulted in a decrease in the amplitude. The increase in amplitude due to increased p53 turnover is expected, since an increase in protein synthesis enables p53 (and consequently Mdm2), to reach higher levels before the feedback kicks in to limit its abundance. An increase in the binding affinity of Mdm2 to p53, results in more p53 being targeted for degradation, which explains the predicted decrease in amplitude. The predicted decrease in amplitude due to an increase in the turnover of Mdm2_mRNA is less intuitive but can be explained as follows. An increase in the turnover of Mdm2_mRNA increases the responsiveness of the system, resulting in a more rapid increase in Mdm2_mRNA and hence Mdm2 protein synthesis following DNA damage. This increase in Mdm2 leads to some restoration of p53/Mdm2 binding and so p53 levels decrease and the oscillation amplitude is thereby lowered.

### (2) ATM Model

#### Variable oscillations after DNA damage

**Figure 9:**
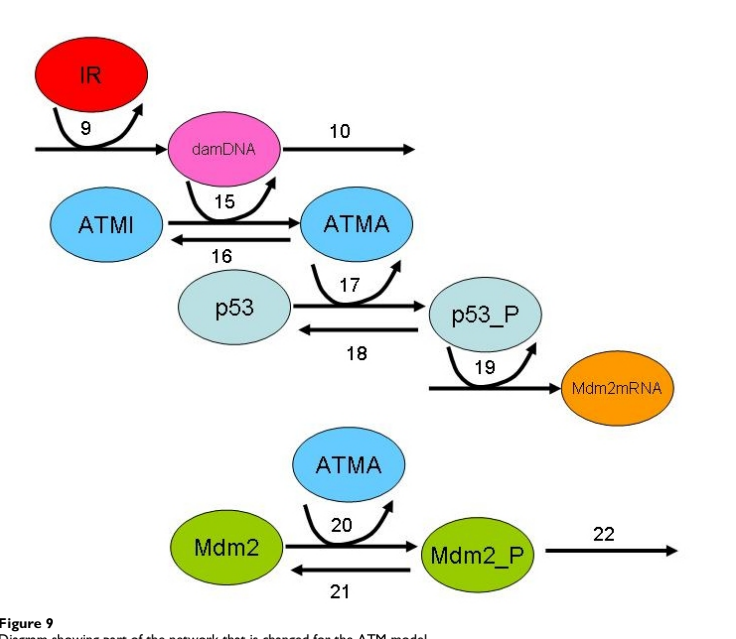
Diagram showing part of the network that is changed for the ATM model.

**Figure 10:**
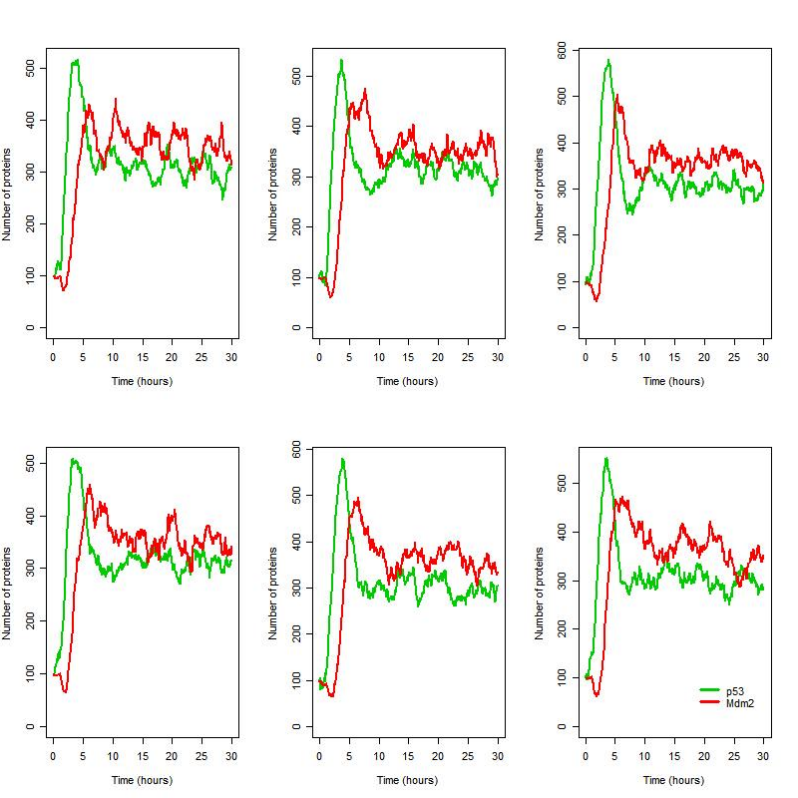
Six simulations for the ATM model under conditions of irradiation (IR = 25 dGy for 1 minute at time t = 1 hour).

Figure 9 shows the part of the ATM model which differs from the ARF model. We found that this model generally produced one large initial peak of p53 after DNA damage, followed by irregular oscillations in which p53 levels remained relatively high (Figure 10).

#### Addition of p53_mRNA

**Figure 11:**
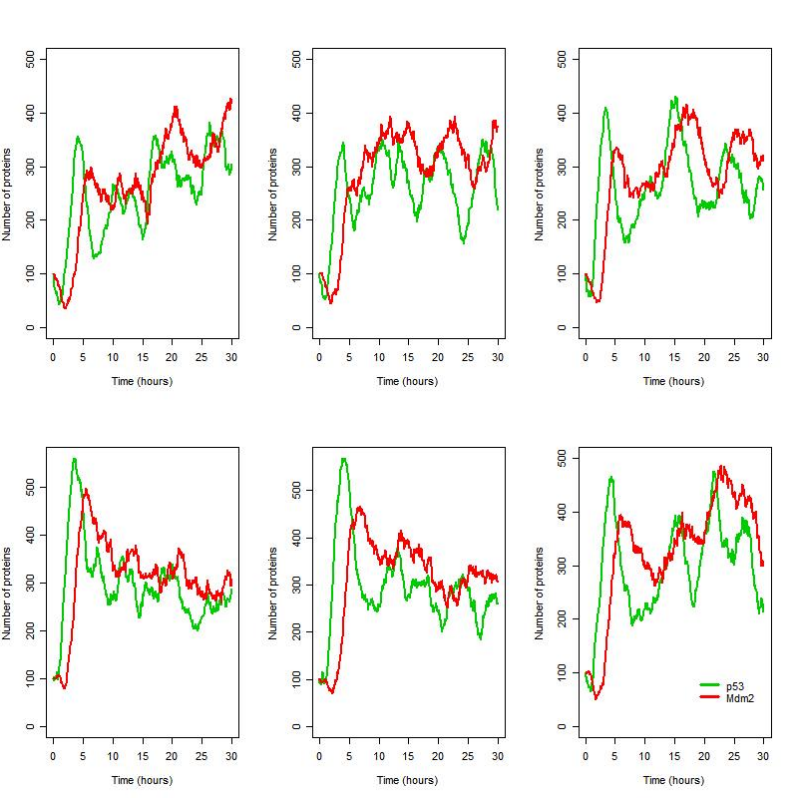
Six simulations for the ATM model under conditions of irradiation (IR = 25 dGy for 1 minute at time t = 1 hour) with an additional step for p53 synthesis.

The reaction for p53 synthesis was modified by adding in a step for transcription so that p53 synthesis depended on the level of p53_mRNA. The addition of this step resulted in an increase in noise in p53 synthesis. The model now predicts sustained oscillations with considerable intercellular variability as seen in the experimental data (Figure 11).

#### Effect of varying model parameters

As for the ARF model we varied the parameters to see if the oscillatory behaviour was sensitive to the changes in their values. The model was fairly robust to parameter changes for a decrease or increase by one order of magnitude. Similar to the ARF model, a ten-fold decrease in p53 turnover resulted in loss of oscillations. In the same way that the rate of ARF activation had little effect on the model outcome, varying the rate of ATM activation had no effect on the period or amplitude of oscillations. Varying the parameter for p53 phosphorylation, resulted in a longer period and higher levels of p53 and Mdm2 when the rate was increased ten-fold, but a ten-fold decrease had little effect. The parameter for Mdm2 phosphorylation was much more sensitive with a ten-fold increase leading to loss of oscillations and a ten-fold decrease resulting in a much lower amplitude.

## Discussion

We have developed two simple stochastic models of the p53/Mdm2 circuit that reflects biological reality with the minimum of detail. We considered two different mechanisms for the DNA damage response: ARF activation, followed by sequestering of Mdm2, and ATM activation followed by phosphorylation of Mdm2 and p53. The reason that we chose to model both mechanisms is to encompass scenarios in which cells do or do not express ARF. For some experiments H1299 human lung cancer cells (which have been shown to be ARF positive [44]) may be utilized, but the current experimental data comes from cells which do not express ARF [30].

We have started from the biology and built network models that incorporate biological mechanisms and then by using SBML have converted these into discrete stochastic models that can be simulated. Both models contain one negative feedback loop and all the reactions contain rates that are based on mass action kinetics. The first model was based on the p14^ARF ^mechanism of sequestering Mdm2 after DNA damage which has the effect of stabilising p53 and increasing its transcriptional activity. If there is sustained DNA damage, this model produces oscillations which closely match experimental data in terms of the period, amplitude, intracellular variability between peaks and intercellular variability. A common criticism of mathematical models is that they are very dependent on the choice of parameter values particularly where values are not available from experimental data but have to be estimated by model fitting. However, in this model the behaviour was robust to changes in the parameter values and it predicted that oscillations were only lost when there was insufficient or unsustained DNA damage, a ten-fold decrease in p53 turnover or a ten-fold increase in Mdm2 turnover. Only a few of the parameters affect the period or amplitude of the oscillations, and these are mainly the parameters involved with p53 and Mdm2 turnover. These are the parameters for which we have the most certain information since the half-life of both p53 and Mdm2 is known to be approximately 20–30 minutes [12].

Our model suggests that oscillations appear after DNA damage due to the action of p14^ARF ^binding to Mdm2. This interferes with p53-Mdm2 binding and prevents p53 degradation, so that p53 levels are able to rise. This is followed by a rise in Mdm2 and when pools of Mdm2 are high, there is sufficient Mdm2 to allow p53 binding to resume. This results in p53 levels decreasing, and with less p53, levels of Mdm2 also start to decline. If pools of ARF are still active when levels of Mdm2 are lowered, p53 is prevented from binding to Mdm2 and the cycle begins again. The oscillations continue as long as there is sufficient p14^ARF ^to compete with p53 for Mdm2 binding. Therefore oscillations are only observed when there is a DNA damage signal. If p53 turnover is too slow, then p53 is unable to rise to sufficient levels to allow oscillations. On the other hand if Mdm2 turnover is too rapid, then there is always enough Mdm2 present to bind to p14^ARF ^and p53. Therefore our model predicts that the appearance of oscillations is dependent on the relative amounts of p53, Mdm2 and p14^ARF ^present in the cell, and it might be instructive to look at flow cytometric measurements of these proteins. The predictions of our model are amenable to experimental confirmation through genetic manipulation of p53, Mdm2, and p14^ARF ^levels within cells exposed to DNA damage.

p53 is important for eliminating cells with irreparable DNA damage to preclude the growth of tumours, but this mechanism would not apply to postmitotic neurons; nevertheless p53 oscillations may still result in neuronal loss. There is evidence to suggest that loss of p53 improves the outcome in models of the expanded polyglutamine disorders Huntington's disease and spinocerebellar ataxia type 1 [45-47]. The accumulation of abnormal protein may induce p53 oscillations through generation of ROS [48,49]. Our simulations indicate that an age-related accumulation of ROS may trigger p53/Mdm2 oscillations at a time in life when proteasome efficiency is declining. With regard to the brain (where the most dramatic decline in proteasome activity has been documented [43,50-52]) it would therefore seem particularly important to understand how oscillations are affected by alterations in the ubiquitin/proteasome system.

The mechanism of p14^ARF ^binding to Mdm2 has some similarities with the ATM mechanism. ATM phosphorylates Mdm2 and p53 which disrupts their ability to form a complex. ATM also enhances the degradation of Mdm2. Wagner et al used a deterministic model to show that ATM induces oscillations by increasing feedback strength and effective dampening [53].

The variability in the oscillations is due the stochastic nature of the processes in the model, especially the amount of DNA damage and the rate of its repair. We propose that this would account for the intercellular variability in the number of peaks. Since the protein synthesis and degradation is also inherently stochastic, the relative amounts of p53, Mdm2, and p14^ARF ^also vary with time and we hypothesise that this accounts for the intracellular variability in the oscillation amplitude.

Our models show that oscillations are very robust to parameter changes when we assume that the mechanism for p53 stabilization is via ARF. This shows that the close agreement between model predictions and experimental data is not a result of our choice of parameter values. In fact most of the parameters are based on known values and the parameters for which we were least confident had the smallest effect on the model behaviour.

We also modified the model so that the mechanism for p53 stabilization is phosphorylation by activated ATM. We have found that a model based on this mechanism requires an additional step for p53 synthesis and produces very variable oscillations. The experimental data [30] also shows large variations in the oscillatory behaviour and since the MCF7 cells which were used did not express ARF, our model agrees with the data. It would be interesting to repeat the experiments carried out in Uri Alon's laboratory on a cell line which expresses ARF to see if more regular sustained oscillations are obtained. Our models may help to explain why oscillations are only observed in certain cell types.

We used stochastic simulation as this is the most natural way to introduce the cellular variability which is seen experimentally. Also some of the species, such as the amount of DNA damage, have low values which necessitate a stochastic approach.

**Figure 12:**
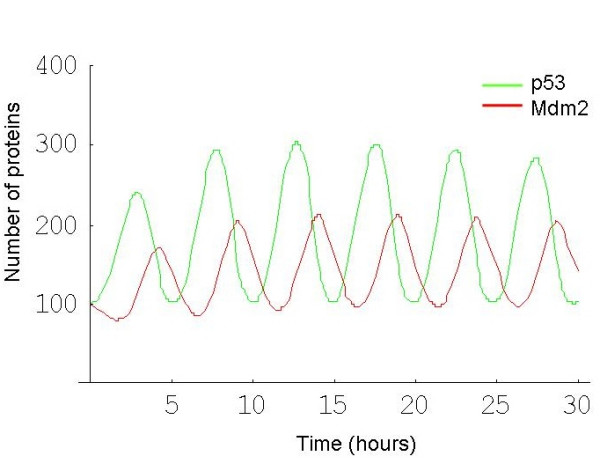
Deterministic solution for the ARF model.

**Figure 13:**
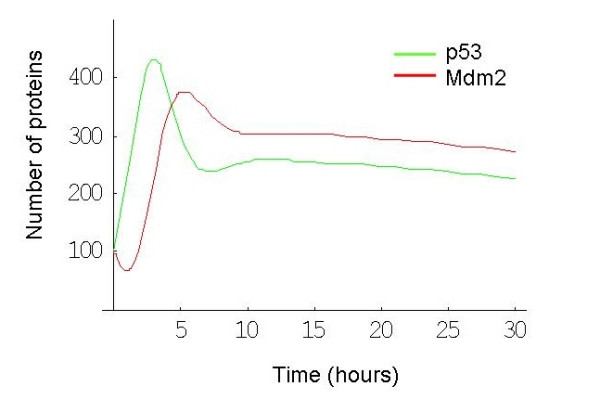
Deterministic solution for the ATM model.

In order to show that the variability in the oscillations is qualitatively different from those seen in deterministic simulations, we also performed deterministic simulations on the models (Figures 12 and 13). In the ARF model, oscillations are still produced but interestingly, the deterministic version of the ATM model predicts only one peak followed by fairly constant levels of total p53 and Mdm2 but at a level higher than the initial values. The lack of oscillations may be either due to the averaging effect since there is variability in the period in this model, or due to the approximation used to derive the reaction rate equations in the deterministic simulation. We carried out 1000 repeat simulations of the stochastic ATM model and compared the mean with the deterministic simulation. We found that the plot of the mean was very similar to the deterministic output, confirming that the lack of oscillations was due to the averaging effect (data not shown). Therefore the stochastic model shows stochastic oscillations consistent with the data for single cell measurements, whereas the deterministic model loses the oscillations due to averaging out effects. The averaging effect is due to the inter-cell variability in the oscillatory period and although the cells are synchronised for the first peak, they are unsynchronised for all the following peaks. Therefore the oscillations in the different cells cancel out. This is also observed experimentally if measurements are taken for a population of cells rather than individual cells [35].

We are currently collaborating with members of Newcastle School of Mathematics and Statistics to fit the model to experimental data using new Bayesian calibration techniques. This work will also shed more light on whether the ARF or ATM mechanism is most likely to account for the variability in the oscillatory behaviour.

SBML is a well known modelling standard that allows models to be shared and easily modified as new data emerges. The SBML code is available [see Additional file 1 for the ARF model and Additional file 2 for the ATM model]. It can also be downloaded from the BASIS system or the Biomodels database [41,54]. An additional advantage of using SBML is that the model can be easily embedded into a larger model of the ubiquitin-proteasome system and linked to models of DNA damage and telomere-dependent checkpoint pathways that are being developed within the Centre for Integrated Systems Biology of Ageing and Nutrition at Newcastle University. This work is currently in progress.

## Conclusion

We have developed models of the p53-Mdm2 circuit to examine the cellular mechanisms that might contribute to the variability in the pattern of sustained oscillations after DNA damage. We considered that stabilisation of p53 can occur either through activation of p14^ARF ^or via activation of ATM. The models predict more regular oscillations if ARF is present and suggest the need for further experiments in ARF positive cells to test these predictions. The models were encoded in SBML to ensure that they can be easily modified and extended as more data become available. Our work illustrates the importance of systems biology approaches to understanding the complex role of p53 in both ageing and cancer.

## Methods

### Construction of network models

We assume that p53 is synthesised at a constant rate, and that under normal conditions it is usually bound to Mdm2 and then degraded. We assume that p53 is only transcriptionally active when not bound to Mdm2, and so the production of Mdm2_mRNA is dependent on the pool of unbound p53. The synthesis of Mdm2 depends on the level of Mdm2_mRNA and so is also dependent on the level of unbound p53. Thus Mdm2_mRNA provides the intermediary link between p53 and Mdm2 to provide the necessary delay in the negative feedback loop. We also include degradation of Mdm2 and Mdm2_mRNA.

To carry out a 'virtual experiment' whereby the cell is subject to irradiation, we use an SBML event structure in the model so that after the simulation has been running for 1 hour (virtual time) the level of a species named IR goes up from 0 to 25 dGy for 1 minute and then returns back to zero. A reaction to mimic damage to DNA depends on the level of IR and only occurs when IR > 0 dGy. A species called damDNA keeps track of the amount of damaged DNA in the cell. We assume that damaged DNA can be repaired at rate *k*_*repair*_.

### ARF model

The presence of damaged DNA activates ARF which increases from zero with rate *k*_*actARF *_<# damDNA>, where <#damDNA> denotes the number of damaged DNA molecules. We assume that ARF binds to Mdm2 with a higher affinity than p53 and so levels of unbound p53 increase. This results in an increase of p53 transcriptional activity and so we predict an increase in levels of Mdm2_mRNA, followed by an increase in Mdm2. Since it is known that ARF increases the degradation rate of Mdm2, we assume that Mdm2 which is bound to ARF is degraded at a higher rate than normal. ARF is also degraded which allows the damage signal to decline as the damaged DNA is repaired.

A graphical representation of the model is given in Figure 3. A list of all the components of the model (which we refer to as 'species' using the terminology of SBML) is given in Table 1. Note that the initial values for p53 and Mdm2 refer to the number of unbound molecules and that the majority of p53 is bound to Mdm2 in the Mdm2_p53 complex. So initially we assume that there are 100 molecules of p53 and Mdm2. A list of all the interactions between the species (termed 'reactions') and the kinetic parameters involved in each reaction are shown in Table 2.

### ATM Model

The ATM model contains the same species as the ARF model except that ARF and ARF_Mdm2 were removed and five new species were added to represent p53_mRNA, phosphorylated p53, phosphorylated Mdm2, and ATM in its inactive and active form (see Table 4). The reactions involving ARF were removed (i.e. reactions 11–14 of Table 2 and replaced with reactions for phosphorylation of p53 and Mdm2, ATM activation and inactivation, and p53_mRNA turnover (see Table 5). We also modified the step for p53 synthesis by adding in steps for transcription. This addition to the model had the effect of adding additional noise to p53 synthesis.

**Table 4:** Additional species for the ATM model. (ARF and ARF_Mdm2 were removed)

Name	Description	Database term	Initial amount
p53_P	Phosphorylated p53	P04637	0
Mdm2_P	Phosphorylated Mdm2	Q00987	0
ATMI	Inactive ATM	Q13315	200
ATMA	Active ATM	Q13315	0
p53_mRNA	p53 messenger RNA	SBO:0000278	20

Terms starting with:

P or Q are from UniProtKB/Swiss-Prot [61]

SBO are from Systems Biology Ontology [62]

**Table 5:** List of changed and additional reactions for the ATM model

Reac No.^a^	Name	Term	Kinetic law	Parameter values^b^	
1	p53 synthesis	GO:0006412	*k*_*synp*53 _<#p53_mRNA>	6.0E-3 s^-1^	Based on p53 half-life ~20 min[12]
15	ATM activation	GO:0006468	*k*_*actATM*_<#damDNA><#ATMI>	1.0E-4 mol^-1 ^s^-1^	Phosphorylation takes place on time-scale of a few minutes. Rate of reaction depends on amount of damaged DNA and pool of ATMI.
16	ATM inactivation	GO:0006470	*k*_*inactATM*_<#ATMA>	5.0E-4 s^-1^	De-phosphorylation is of the same order of magnitude as phosphorylation.
17	p53 phosphorylation	GO:0006468	*k*_*phosp*53 _<#p53><#ATMA>	5.0E-4 mol^-1 ^s^-1^	Phosphorylation takes place on time-scale of a few minutes.
18	p53 dephosphorylation	GO:0006470	*k*_*dephosp*53 _<#p53_P>	5.0E-1 s^-1^	This parameter was varied to obtain oscillations.
19	Mdm2 Transcription	GO:0003700	*k*_*synMdm*2*mRNA*_<#p53_P>	1.0E-4 s^-1^	Same as ARF model
20	Mdm2 phosphorylation	GO:0006468	*k*_*phosMdm*2_<#Mdm2><#ATMA>	2.0 mol^-1 ^s^-1^	This is more rapid th.an phosphorylation of p53 by ATM [22].
21	Mdm2 de-phosphorylation	GO:0006470	*k*_*dephosMdm*2 _<#Mdm2_P>	5.0E-1 s^-1^	De-phosphorylation is of the same order of magnitude as phosphorylation.
22	Mdm2 degradation enhanced by ATM	GO:0043161	*k*_*degMdm*2*ATM*_<#Mdm2_P>	4.0E-4 s^-1^	Mdm2 is degraded at higher rate after phosphorylation by ATM[21].
23	p53_mRNA synthesis	GO:0009299	*k*_*synp*53*mRNA*_	1.0E-3 mol s^-1^	Assume turnover of mRNA is about 2 hours
24	p53_mRNA degradation	GO:0006402	*k*_*degp*53*mRNA*_<#p53_mRNA>	1.0E-4 s^-1^	

^a^Reaction numbers correspond to the numbered arrows in Figures 3 and 9, ^b^mol refers to the number of molecules

### Parameter Values

We used mass action kinetics for all the reaction rates [55]. The last column in Tables 2 and 5 gives details and references for the chosen parameter values. Most of the parameter values were taken from the literature and we can be fairly confident of their values. In particular we were confident of the values of the parameters which had most effect on the model behaviour such as p53 and Mdm2 turnover rates. The parameters that we were least confident about were the rate of ARF binding to Mdm2 and the rate of ARF-dependent Mdm2 degradation. However the model was robust to changes in either of these parameters.

We chose default values for all the parameters involved in p53 and Mdm2 turnover which would give a half-life of about 20 minutes for p53 and 30 minutes for Mdm2 under normal conditions. The binding rate of p53 to Mdm2 was set so that about 95% of the pool of p53 is bound to Mdm2 under normal conditions and the dissociation rate was set to 100 molecules which corresponds to about 277 nM, assuming the cell volume to be 0.6 μm^3^. This is within the range 60–700 nM suggested by experimental measurements [56]. We then included the event for stressing the cell and adjusted the parameter values for Mdm2_mRNA turnover until we obtained a period of about 5.5 hours for the oscillations and a delay of about 2 hours between the p53 and Mdm2 peaks. We carried out sensitivity analysis by varying each parameter over a range of ×0.1 to ×10.0 of the original value. In some cases we varied combinations of parameters simultaneously, since some of the parameters are not independent. For example, we looked at varying the turnover rate of p53 and Mdm2 by varying the synthesis and degradation rates together, so that the total levels of the proteins remained constant.

### Model code and analysis of results

We used SBML to translate the graphical model into a computer readable format. The model was first encoded using SBML shorthand [55] and then translated into full SBML and imported into the BASIS modelling environment using web-services [57]. Simulations were carried out using the stochastic simulator which is based on the Gillespie algorithm [58]. The SBML code can be accessed by the reader [see Additional file 1 for the ARF model and Additional file 2 for the ATM model]. The models are also available in the public space of BASIS (ARF model: urn:basis.ncl:model:4770; ATM model: urn:basis.ncl:model:4775) and the Biomodels database [54,59] [ARF Model:MODEL:8142536273, ATM model: MODEL:5836973167] and can be freely accessed by the reader. Repeat simulations were carried out to investigate the variability in the model output. The model output was exported from BASIS to the R statistical package, an open-source software application, and the results were analysed and plotted. The autocorrelation function was also plotted for each result to confirm whether the oscillations were distinct from white noise. The deterministic simulations were performed in Mathematica using MathSBML [60].

## Authors' contributions

CJP constructed and coded the models, ran simulations, plotted and analysed results and drafted the manuscript. DAG advised on the model construction and contributed sections of the manuscript. Both authors read and approved the final manuscript.

## Supplementary Material

Additional file 1 Proctor_p53_Mdm2_ARF. SBML code for the p53/Mdm2 ARF model. Click here for file

Additional file 2 Proctor_p53_Mdm2_ATM. SBML code for the p53/Mdm2 ATM model. Click here for file

Additional file 3 p53paper. Endnote library. Click here for file
